# Colonic Perineurioma Presenting as a Small Subepithelial Lesion With Distinctive Endoscopic Findings

**DOI:** 10.1002/deo2.70361

**Published:** 2026-06-09

**Authors:** Hironobu Takedomi, Moeko Shirozu, Kayoko Fukuda, Takashi Yao, Motohiro Esaki

**Affiliations:** ^1^ Department of Internal Medicine Division of Gastroenterology Faculty of Medicine Saga University Saga Japan; ^2^ Department of Gastroenterology Hiramatsu Hospital Saga Japan; ^3^ Department of Human Pathology Juntendo University Graduate School of Medicine Tokyo Japan

**Keywords:** colonoscopy, differential diagnosis, pathology, perineurioma, subepithelial lesion

## Abstract

A 59‐year‐old man with a history of colorectal polypectomy underwent a screening colonoscopy. A 5‐mm reddish lesion was identified in the sigmoid colon. The lesion had a smooth surface without erosion or ulceration and appeared slightly elevated with well‐demarcated margins. Magnifying narrow‐band imaging revealed dilated round pits, reduced crypt density, and arborizing vessels in the central area. Cold snare polypectomy was performed for diagnostic and therapeutic purposes. Histopathological examination demonstrated proliferation of bland spindle cells with oval nuclei in the lamina propria. Immunohistochemistry showed positivity for vimentin and GLUT‐1, weak epithelial membrane antigen expression, and negativity for S‐100, SMA, c‐kit, and CD34, with a Ki‐67 index of <1%. These findings were consistent with colonic perineurioma, a rare benign peripheral nerve sheath tumor composed of perineurial cells. This case highlights that colonic perineurioma should be considered in the differential diagnosis of small subepithelial lesions detected during colonoscopy. Reduced crypt density and arborizing vessels may provide subtle clues; however, definitive diagnosis requires histopathological and immunohistochemical evaluation.

## 1 Introduction

Perineurioma is a rare benign peripheral nerve sheath tumor composed of perineurial cells [1]. With an estimated incidence of 0.1%–1.46% of all colonic polyps, colonic perineurioma is an uncommon lesion that may be detected incidentally in asymptomatic patients undergoing screening colonoscopy, most often in the distal colon [2, 3]. However, reports remain limited, particularly regarding its endoscopic features. Herein, we report a case of colonic perineurioma with distinctive endoscopic findings.

## 2 Case Report

**FIGURE 1:**
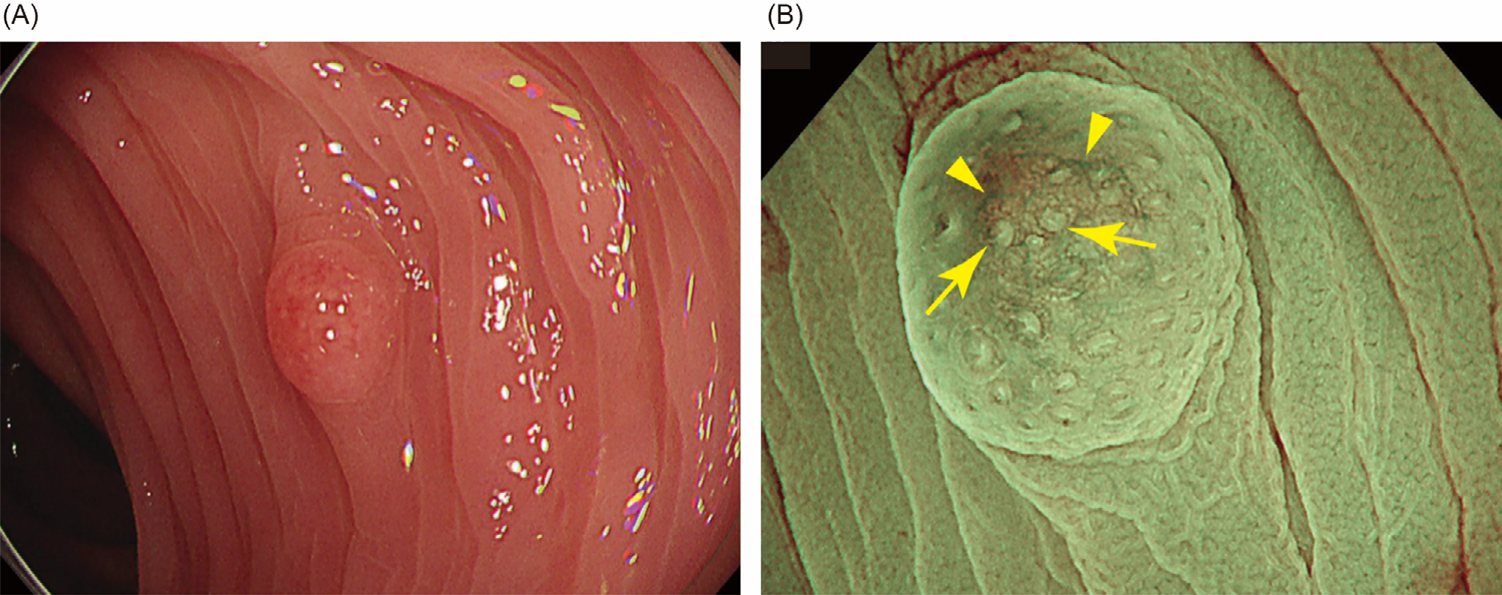
Endoscopic findings of a perineurioma in the sigmoid colon. (A) Colonoscopy revealed a 5‐mm reddish sessile lesion with a smooth surface and well‐demarcated margins. (B) Magnifying narrow‐band imaging shows dilated round pits with reduced crypt density (arrow) and arborizing vessels (arrowhead) in the central area.

**FIGURE 2:**
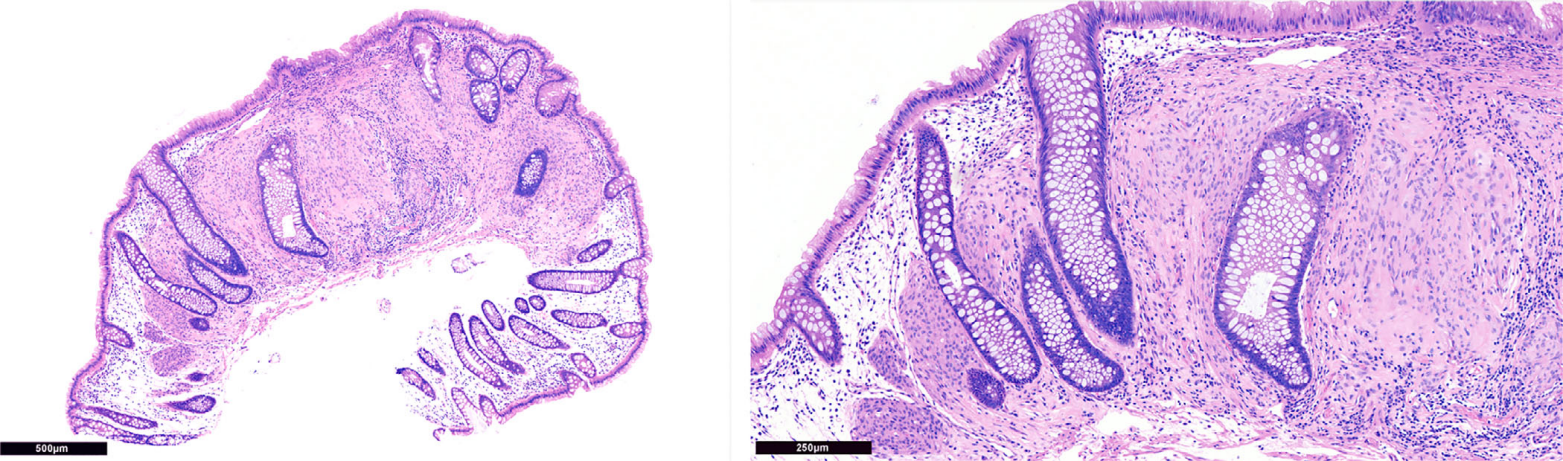
Histopathological findings. Hematoxylin–eosin staining shows proliferation of bland spindle cells with oval nuclei in the lamina propria. (scale bars: right, 500 µm; left, 250 µm).

**FIGURE 3:**
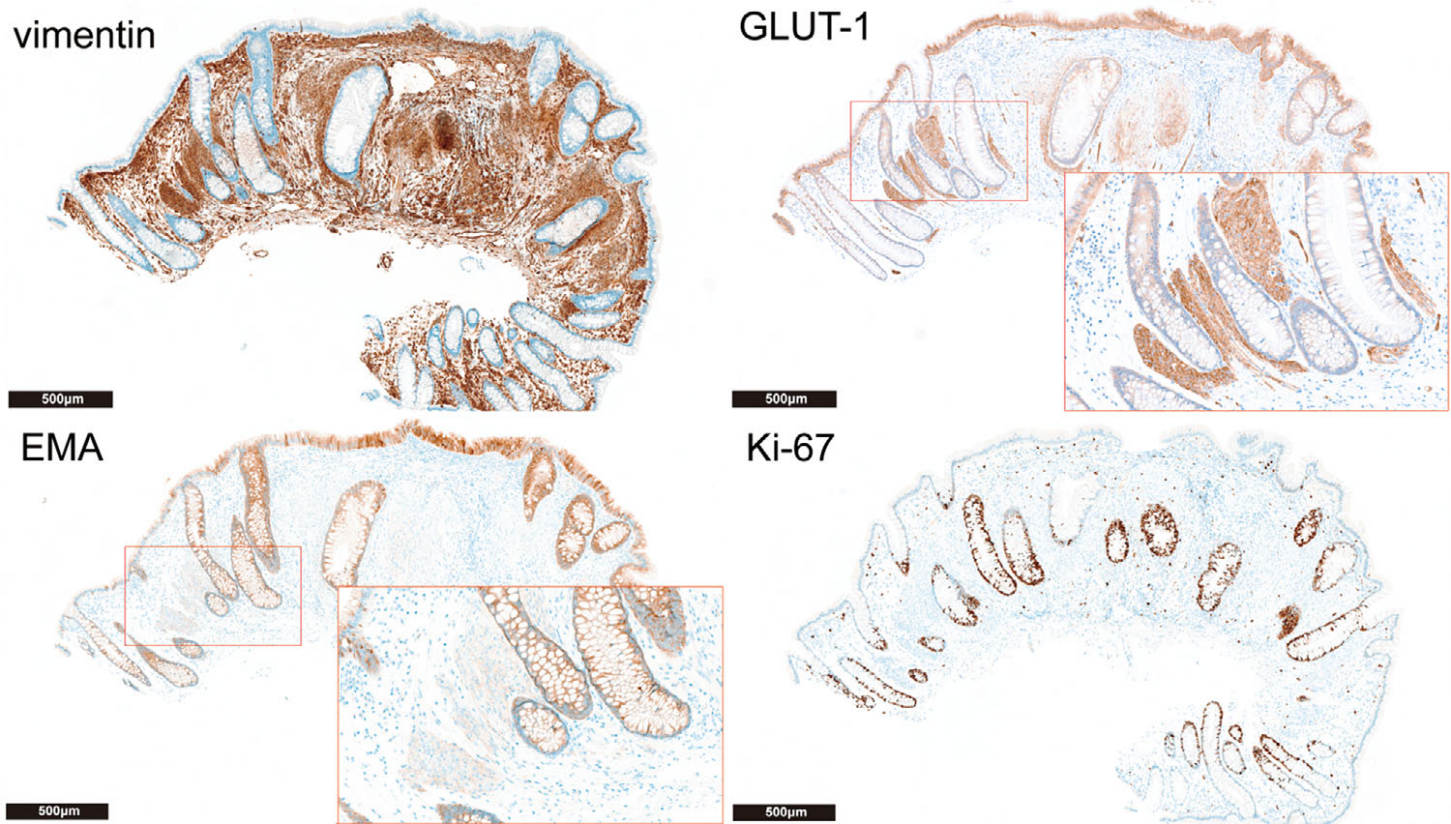
Immunohistochemical findings. Immunohistochemical analysis demonstrates positivity for vimentin and GLUT‐1, weak positivity for epithelial membrane antigen (EMA), and a Ki‐67 index of <1%. (scale bar: 500 µm).

A 59‐year‐old man with a history of colorectal polypectomy underwent a screening colonoscopy. He was asymptomatic at presentation. Colonoscopy revealed a 5‐mm reddish lesion in the sigmoid colon. The surface was smooth without erosion or ulceration, and the lesion appeared slightly elevated with well‐demarcated margins from the surrounding mucosa (Figure 1A). Magnifying observation with narrow‐band imaging (NBI) demonstrated dilated round pits, along with reduced crypt density and arborizing vessels in the central area (Figure 1B). Although the endoscopic findings were atypical, a sessile serrated lesion could not be excluded; therefore, cold snare polypectomy was performed for diagnostic and therapeutic purposes. Histopathological examination revealed proliferation of bland spindle cells with oval nuclei in the lamina propria (Figure 2). Immunohistochemistry showed positivity for vimentin and GLUT‐1, weak epithelial membrane antigen (EMA) expression, and negativity for S‐100, SMA, c‐kit, and CD34. The Ki‐67 index was <1% (Figure 3 and Figure S1). These findings were consistent with a diagnosis of perineurioma.

## 3 Discussion

Colonic perineurioma is a rare benign peripheral nerve sheath tumor composed of perineurial cells, characterized by proliferation of spindle cells with perineurial differentiation in the lamina propria [1, 2]. It was first described by Eslami‐Varzaneh et al. in 2004 as a benign fibroblastic polyp (BFP) [4]. Based on shared histopathological and immunohistochemical features, BFP is now regarded as the same entity as perineurioma.

Perineuriomas in the gastrointestinal tract have been reported primarily in case reports and small case series rather than in large‐scale epidemiological studies. A recent systematic review reported that the mean age of patients with colonic perineurioma was approximately 52 years (range 24–87 years), with a slight female predominance (58.7%) [2]. These lesions are most commonly located in the distal colon, particularly in the sigmoid and rectosigmoid regions. Most colonic perineuriomas are asymptomatic and are incidentally detected during routine screening colonoscopy. To date, no recurrence or metastasis has been reported, and perineurioma is therefore regarded as a benign tumor.

Although the endoscopic characteristics of colonic perineurioma have not been fully established, these lesions are generally described as solitary small sessile polyps or submucosal tumor (SMT)‐like lesions in the distal colon and may exhibit endoscopic features overlapping with those of hyperplastic polyps or sessile serrated lesions, even in the absence of an associated serrated epithelial component [2, 3]. Previous reports describing magnifying endoscopic findings of colonic perineurioma are limited, although dilated crypt openings, reduced crypt density, and dendritic or arborizing vascular patterns have been described in a small number of cases [5, 6]. Some of these findings, including sessile morphology and dilated crypt openings, may overlap with those observed in sessile serrated lesions. In contrast, the arborizing vessels and reduced crypt density observed in the present case appear atypical for conventional sessile serrated lesions. These findings may reflect underlying spindle cell proliferation within the lamina propria and may differ from the more uniform crypt architecture typically observed in sessile serrated lesions.

Immunohistochemically, perineurioma shows positivity for EMA, GLUT‐1, and claudin‐1, reflecting perineurial differentiation, whereas S‐100 is negative, distinguishing it from schwannoma. Negativity for α‐SMA, desmin, c‐kit, and CD34 helps exclude leiomyoma, gastrointestinal stromal tumor, and inflammatory fibroid polyp. A low Ki‐67 index supports its benign nature [5, 7]. It has been reported that intramucosal perineuriomas are frequently associated with serrated epithelial components, suggesting a possible epithelial–stromal relationship [8]. However, no serrated epithelial component was identified in the present case.

This case highlights that colonic perineurioma should be considered in the differential diagnosis of small subepithelial lesions, especially in the distal colorectum, detected during colonoscopy. Reduced crypt density and arborizing vessels may provide subtle clues; however, definitive diagnosis requires histopathological and immunohistochemical evaluation. Given the limited data regarding its endoscopic features, further accumulation of well‐characterized cases is warranted to better characterize its endoscopic and clinical significance.

## Author Contributions

**Conceptualization and data curation**: Hironobu Takedomi. **Investigation**: Hironobu Takedomi, Moeko Shirozu, Kayoko Fukuda, and Takashi Yao. **Supervision**: Motohiro Esaki. **Visualization**: Hironobu Takedomi and Takashi Yao. **Project administration**: Hironobu Takedomi and Motohiro Esaki. **Resources**: Hironobu Takedomi, Moeko Shirozu, Kayoko Fukuda, and Takashi Yao. **Writing – original draft**: Hironobu Takedomi. **Writing – review & editing**: Moeko Shirozu, Kayoko Fukuda, Takashi Yao, and Motohiro Esaki.

## Funding

The authors have nothing to report.

## Ethics Statement

This study was conducted in accordance with the Declaration of Helsinki.

## Consent

Informed consent was obtained from the patient.

## Conflicts of Interest

Motohiro Esaki is a Deputy Editor‐in‐Chief of DEN Open. The other authors declare no conflicts of interest.

## Supporting information




**Supporting Figure 1**: Immunohistochemical findings. Immunohistochemical analysis demonstrates negativity for S‐100, SMA, CD34, and c‐kit (scale bar: 500 µm).
