# Supplementary figures and images for: Colonic Perineurioma Presenting as a Small Subepithelial Lesion With Distinctive Endoscopic Findings
Source: DEN Open. 2026 Jun 9;7(1):e70361. doi: 10.1002/deo2.70361 (PMC13250387; doi:10.1002/deo2.70361)

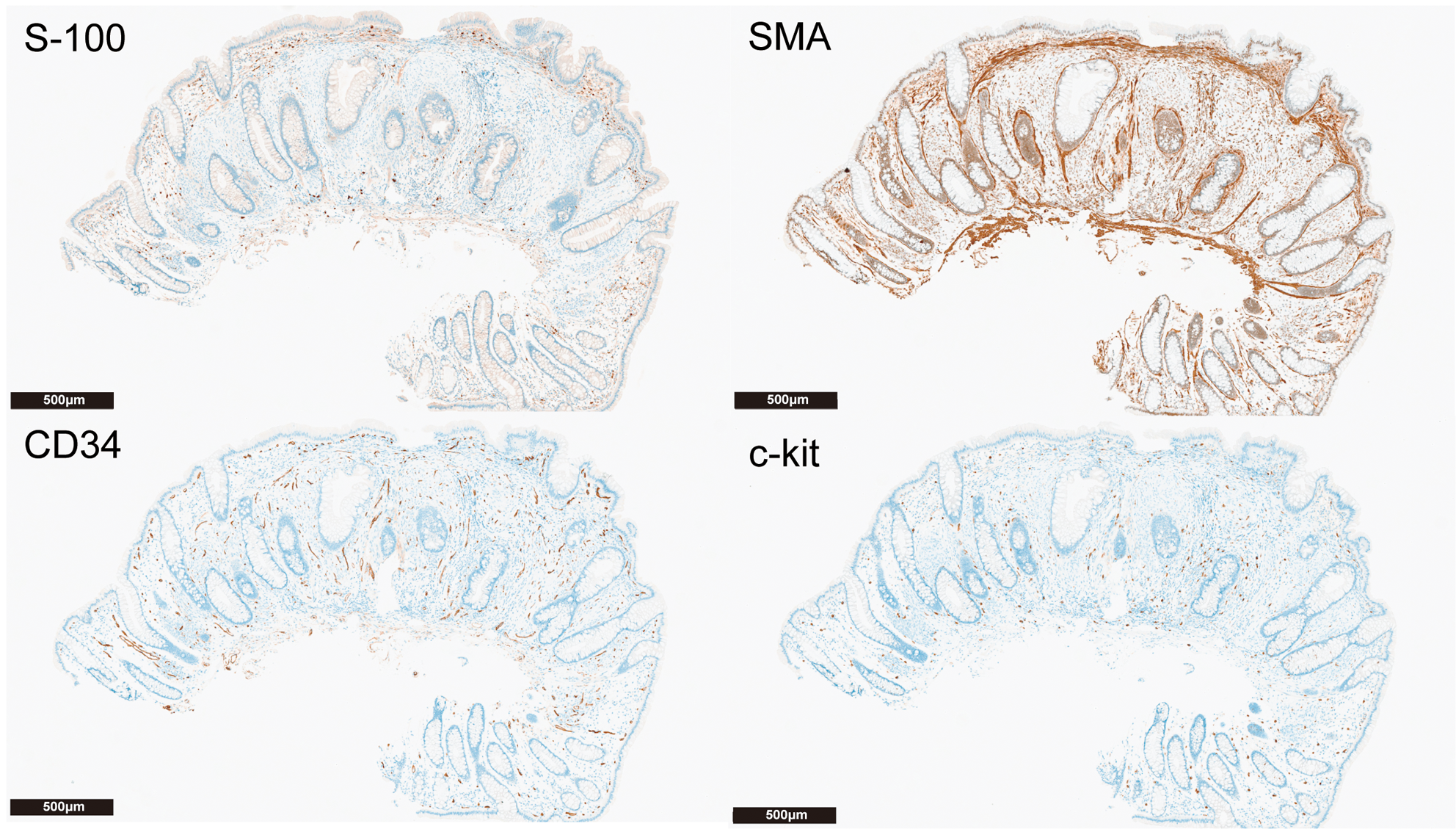

Supplement: Supplementary file 1 — Supporting Figure 1: Immunohistochemical findings. Immunohistochemical analysis demonstrates negativity for S‐100, SMA, CD34, and c‐kit (scale bar: 500 µm). [file DEO2-7-e70361-s001.tif]
